# Psychometric evaluation of the SF-36 (v.2) questionnaire in a probability sample of Brazilian households: results of the survey Pesquisa Dimensões Sociais das Desigualdades (PDSD), Brazil, 2008

**DOI:** 10.1186/1477-7525-9-61

**Published:** 2011-08-03

**Authors:** Josué Laguardia, Monica R Campos, Claudia M Travassos, Alberto L Najar, Luiz A Anjos, Miguel M Vasconcellos

**Affiliations:** 1 Laboratório de Informação em Saúde, Instituto de Comunicação e Informação Científica e Tecnológica em Saúde, Fundação Oswaldo Cruz, Av. Brasil 4356, Pavilhão Haity Moussatché sala 214, Manguinhos, Rio de Janeiro, Brazil; 2 Departmento de Ciências Sociais, Escola Nacional de Saúde Pública, Fundação Oswaldo Cruz, Av. Leopoldo Bulhões 1480 Manguinhos, Rio de Janeiro, Brazil; 3 Departamento de Nutrição Social, Universidade Federal Fluminense, Rua Mário Santos Braga 30, Valonguinho, Niterói, Brazil

## Abstract

**Background:**

In Brazil, despite the growing use of SF-36 in different research environments, most of the psychometric evaluation of the translated questionnaire was from studies with samples of patients. The purpose of this paper is to examine if the Brazilian version of SF-36 satisfies scaling assumptions, reliability and validity required for valid interpretation of the SF-36 summated ratings scales in the general population.

**Methods:**

12,423 individuals and their spouses living in 8,048 households were selected from a stratified sample of all permanent households along the country to be interviewed using the Brazilian SF-36 (version 2). Psychometric tests were performed to evaluate the scaling assumptions based on IQOLA methodology.

**Results:**

Data quality was satisfactory with questionnaire completion rate of 100%. The ordering of the item means within scales clustered as hypothesized. All item-scale correlations exceeded the suggested criteria for reliability with success rate of 100% and low floor and ceiling effects. All scales reached the criteria for group comparison and factor analysis identified two principal components that jointly accounted for 67.5% of the total variance. Role emotional and vitality were strongly correlated with physical and mental components, respectively, while social functioning was moderately correlated with both components. Role physical and mental health scales were, respectively, the most valid measures of the physical and mental health component. In the comparisons between groups that differed by the presence or absence of depression, subjects who reported having the disease had lower mean scores in all scales and mental health scale discriminated best between the two groups. Among those healthy and with one, two or three and more chronic illness, the average scores were inverted related to the number of diseases. Body pain, general health and vitality were the most discriminating scales between healthy and diseased groups. Higher scores were associated with individuals of male sex, age below 40 years old and high schooling.

**Conclusions:**

The Brazilian version of SF-36 performed well and the findings suggested that it is a reliable and valid measure of health related quality of life among the general population as well as a promising measure for research on health inequalities in Brazil.

## Background

The use of standardised questionnaires with general health measures provides the opportunity to compare the health profiles of groups with different diagnoses, illness severities, or treatment regimens; to monitor transitions in health status over time [1]; to measure the burden of disease in populations with chronic and psychiatric diseases and in healthy populations; and to compare health outcomes across different health systems [2]. The standardised *Short Form Health Survey 36 *(SF-36) is one of the most common instruments used in health research, both in population-based surveys and in studies to evaluate health policies [3]. Its aim is to detect medically- and socially-relevant differences in health status and changes in health status over time using a small number of statistically-efficient dimensions. For this purpose, a multi-item scale was developed that employed multidimensional health concepts used in comprehensive health surveys, including measures of well-being and self-evaluation of health status [4-6]. The items in the questionnaire were selected from the set of 149 items of the *Functioning and Well-Being Profile*, which covered 40 health concepts used in the *Medical Outcomes Study *(MOS), and organised in a standard version, which is available since 1990 [7]. The *Short Form 36 *(SF-36) consists of 36 questions: one of them measures health transitions over a one-year period and is not used in scale calculation, and the remaining questions are grouped into eight scales or domains. The eight scales can be aggregated into two independent summary measures: physical component summary (PCS) and mental component summary (MCS). Higher scores indicate better health.

The SF-36 was translated into various languages and used in several countries to assess the health perceptions of both the general population and people affected by disease [4,7]. Even though its accuracy is 10% to 20% lower than that of longer questionnaires used in the MOS, its completion time of 5-10 minutes, versatility of use (self-completion, personal or telephone interview with persons aged over 14 years), and levels of reliability and validity above the recommended minimum standards make it an attractive tool for use in combination with other questionnaires in population surveys. Study results show that the SF-36 meets the criteria for data quality and scaling assumptions: the two main components used in the scales -- Physical (PCS) and Mental (MCS) -- explained 74% of the total variance. Experiences using the questionnaire and its reported shortcomings, such as cross-cultural non-equivalence, difficulties with some word meanings, floor and ceiling effects, poor performance of the two Role Function scales and standard layout, were used as a basis for implementing changes in the second version (v.2) of the SF-36, in use since 1996 [8]. These changes included adjusting the layout horizontally, improving the wording of questions to make them less ambiguous, changing the response options of items related to Social and Emotional Functioning from binary to ordinal, eliminating one response option from the Vitality and Mental Health scales, and normalising scale values in order to improve comparability among different groups [4]. The results of studies that used the SF-36 version 2 showed an improvement in accuracy, reliability and validity, without compromising the underlying structure of the conceptual model [6,9].

In Brazil, the SF-36 was used in studies on the quality of life of patients with end stage renal disease undergoing intermittent haemodialysis [10], hypertensive patients [11], patients subjected to surgical repair of hip fracture [12], patients living with HIV/AIDS [13], and in a household survey of residents of the state of São Paulo [14]. In these studies, the scores for SF-36 domains obtained in adult populations showed high reliability and good criterion validity compared to other instruments for assessing quality of life. In 2008, a survey on the social dimensions of inequality named *Pesquisa Dimensões Sociais das Desigualdades *(*PDSD*), coordinated by Instituto Universitário de Pesquisas do Rio de Janeiro (IUPERJ) with the participation of various teaching and research institutions in Brazil (UFMG, UFF, FIOCRUZ, UFRJ, PUC-RJ, UFBA), interviewed people around the country to assess the current situation of the Brazilian society with regard to education, health, and professional paths, with the objective of informing social policies. The Health module of the SSDI evaluated several aspects of health using the standard SF-36 (v.2), whose questions relate to the 4 weeks prior to the interview. Unlike previous applications in the country, which dealt with limited samples of individuals with specific health problems, the PDSD used the SF-36 on a probability sample of Brazilian households, thus estimating national scores to be used in future applications of this instrument. The aim of this paper is to assess whether the scales obtained from the SF-36 (v.2) questionnaire used in the PDSD project meet the minimum psychometric standards of data quality, scaling assumptions, reliability, and validity; reproduce the hypothesised mental and physical dimensions; and the relations between factors and scales predict their associations with external criteria for physical and mental health.

## Methods

### Data source and sampling

The Survey on the Social Dimensions of Inequality (PDSD) was a population-based household survey that interviewed, from July to December 2008, 12,423 heads of households and their spouses living in 8,048 permanent private households in common, non-special areas (including slums) in all regions of Brazil, in both urban and rural settings. The population was divided into sets called domains, defined according to region and setting (urban or rural); 6 domains were established, and the study aimed to obtain indicators for each of them, as well as for the population as a whole. Moreover, since the subject of the study was inequality, a sampling stratum consisting of the richest 10% of each census tract was created in order to improve the accuracy of the indicators of inequality. The sample comprised 1,374 census tracts, divided as follows: 200 in urban areas of the North and Central-West Regions (1,320 households); 336 in urban areas of the Northeast Region (1,776 households); 368 in urban areas of the Southeast Region (1840 households ); 260 in urban areas of the South Region (1,300 households); 60 richest tracts in metropolitan areas (420 households); 54 richest tracts in other areas (432 households); 48 tracts in rural areas of the Northeast Region (480 households); and 48 in other rural areas of the country (480 households). The percentage of households with only one eligible respondent ranged from 96% in rural areas of the Northeast to 31% in the metropolitan region of Rio de Janeiro, and 23% in the richest tracts of metropolitan areas. The estimated number of households in the sample accounted for replacement, in every socioeconomic stratum, due to absence from household or refusal to participate in the study.

Among the households in the initial sample, 571 were ineligible and 20% were replaced, mainly due to the refusal of one spouse to take part in the study or because one of the spouses was not at home during the interview, even though it was scheduled in advance. To circumvent this problem, a pair of interviewers returned to such households during weekends to interview the couples simultaneously in different rooms of the house. In the upper class (wealthier) tracts, apart from the difficulties mentioned above, contact with the subjects was more complicated due to the inaccessibility of buildings and private neighbourhoods (even when not gated) and the difficulty to convince them to answer the questionnaire. As for the collection process, the material produced each day was counted, checked, and filtered by the supervisors; the interviewer was contacted and returned to the field when necessary. After this process, all the questionnaires from each census tract were submitted to the team responsible for collecting field data. The questionnaires were then coded, typed, and had their logical and analytical consistency checked (via SPSS syntax) by a team of 20 researchers who returned to the field when necessary for correction/confirmation. Data entry used automated controls that restricted input only to the valid values for each question. Ten percent of all the material typed was reviewed and stratified according to the 30 data typists, which guaranteed the quality of data entry. The sample size in this study met the International Quality of Life Assessment Project (IQOLA) criteria for comparison between sexes and age groups [15]. Research procedures were in accordance with Helsinki Declaration for protection of human subjects from research risks and consent of research subjects and informants was obtained in advance as mandated by the Code of Ethics of the International Sociological Association.

### Data Collection Instrument

The instrument used in the PDSD included, apart from the Brazilian version of the SF-36 (v.2) [16], questions related to education, work, relationships and housing. The Brazilian version differed from the original questionnaire only in questions 3B, 3G, 3H, and 3I, since bowling and golf are not popular activities in Brazil and because the metric system of units is used in the country. The theoretical model of the SF-36 assumes that the Physical Functioning (10 items), Bodily Pain (2 items), and Role Physical (4 items) scales correlate strongly with the Physical Component and its summary measure (PCS). In turn, the Mental Health (5 items), Role Emotional (3 items), and Social Functioning (2 items) scales correlate more strongly with the Mental Component and its summary measure (MCS). Scales related to physical health are also expected to identify groups of respondents who have physical conditions and to show a lower performance than scales related to mental health in identifying groups with mental conditions. The Vitality (4 items), General Health (5 items) and Social Functioning (2 items) scales should correlate with both components. Thus, scales more focused on the PCS are more sensitive to treatments that target physical diseases, whereas scales more focused on the MCS are more sensitive to drugs and therapies that target mental diseases. The procedures for item recoding, summing the responses for each of the variables that make up the scale, transforming the scales into scores ranging from 0 to 100, and standardisation and normalisation, in which average values vary around value 50 with a dispersion factor of 10, followed the recommendations of the SF-36 developers for calculating the domains [17].

### Data analysis

The socio-demographic characteristics of respondents are described in a frequency table. The completeness, distribution and internal consistency of items and scales were calculated in accordance with methods described in the literature for testing scaling assumptions [7,17]. The internal consistency of items was evaluated by analysis of correlations between the items and their respective scales, applying correction for attenuation in order to correct the effect of adding/subtracting items to/from the estimates [18]. Estimates of internal consistency with values above 0.40 were considered satisfactory. Measures of asymmetry in the distribution of scores and the internal consistency of scales were calculated using Cronbach's alpha coefficient; values greater than 0.70 were taken as the minimum ideal condition for analysis at the group level. In addition, the consistency of responses to the 15 pairs of questions was evaluated, as suggested by the authors of the SF-36 (v.2) [17]. The discriminant validity of items was calculated to assess the integrity of scale construction. For each scale, the success rate was calculated as the ratio of the number of successes to the total number of items tested; a success was counted whenever the correlations between the item and its respective scale were at least two standard errors above the correlations between the same item and the other scales. The percentage of respondents who achieved the highest (ceiling effect) or lowest (floor effect) scores was calculated to assess the instrument's ability to detect changes over time. The equality of item-scale correlations was assessed based on each item's contribution to the total score of the hypothesised scale, and when these correlations ranged from 0.40 to 0.70 it was assumed that the item contributed substantially to the score. The associations between scales and the summary measures of components were calculated using Spearman's correlation coefficients and rotation matrices in factor analysis. Exploratory factor analysis using principal component analysis of the 8 SF-36 scale scores was conducted to extract the hypothesized two components from the correlations among the SF-36 scales. Two factors with eigenvalues greater than 1 were extracted and rotated to orthogonal simple structure using the varimax method to facilitate comparisons with published results and for ease of interpretation. The construct validities of the scales for each component were obtained through the ratio of the squared loading of each scale on the factor and the highest common variance of the respective component. Total, explained and reliable variance were obtained, respectively, from the extraction value of the communalities in each scale and from the division of this value by the scale's Cronbach's alpha. The construct validity of each scale was measured by its ability to detect statistically significant variations in different groups, defined by the presence or absence of chronic disease through the ratio of F-statistic values obtained from the comparison of these groups. The relative validity estimated for each scale was calculated as the ratio of the largest F-value obtained among scales to the F-value of the scale. Data from the heads of households and their spouses were weighted to represent the total Brazilian population. The software SPSS v.17 was used for statistical analysis.

## Results

### Characteristics of the Sample

Among study participants, 5,255 (42.3%) were male, and about half of the respondents were between 40 and 64 years of age (mean: 48.5, SD = 16.0 years), self-classified as white and had more than 4 years of schooling (Table 1). The presence of at least one chronic disease was reported by 63.3% of respondents; the most common conditions were diseases of the vertebral column (36.0%) and hypertension (28.3%). The vast majority (71%) of respondents were married or lived with a partner.

**Table 1:** Descriptive statistics by summary measures of SF-36 v.2, PDSD, 2008

			PCS - Physical component				MCS - Mental component			
				
Variables	N	%	Mean	Min	Max	Median	Mean	Min	Max	Median
*Sex*										

Male	5,255	42.3	50.7	2.4	69.6	54.8	52.9	-1.1	76.0	55.3
Female	7,168	57.7	48.3	5.0	74.7	51.3	49.7	2.5	76.9	52.3
*Age groups (years)*										
18-39	3,973	32.0	54.3	12.7	74.7	57.1	52.1	2.5	75.4	54.5
40-64	6,132	49.4	48.9	5.0	71.0	51.9	50.8	-1.1	76.9	53.4
≥ 65	2,318	18.7	41.8	2.4	63.9	42.1	50.1	5.0	76.5	52.6

Years of schooling*										

0	1,904	16.5	43.2	2.4	68.4	43.5	48.1	7.2	76.0	50.1
1-4	3,592	31.1	47.5	6.3	71.0	50.2	50.7	5.0	74.9	53.2
5-8	2,529	21.9	50.9	5.0	74.7	54.5	51.2	-1.1	76.5	53.8
9-11	2,408	20.9	53.1	11.0	69.8	56.3	52.8	2.5	74.4	55.3
≥ 12	1,109	9.6	53.0	18.9	70.7	56.0	53.1	3.6	73.6	55.3

Race/color (self-atributed)*										

White	5,868	48.7	49.4	2.4	74.7	53.0	51.2	2.9	76.0	53.8
Brown	4,801	39.8	49.3	5.0	72.5	52.7	51.0	-1.1	76.9	53.8
Black	1,389	11.5	49.2	8.6	67.3	52.7	50.9	8.0	74.3	53.2

*Number of chronic conditions*										

0	4,554	36.7	54.9	9.7	71.0	57.5	54.0	5.9	76.9	55.9
1	3,035	24.4	50.3	8.6	74.7	53.4	52.1	2.5	74.4	54.0
2	1,996	16.1	46.4	2.4	70.7	48.3	50.4	3.6	76.5	53.0
≥ 3	2,837	22.8	41.3	6.3	69.8	41.3	45.7	-1.1	75.5	47.4

Total	12,423	100.0	49.3	2.4	74.7	52.9	51.1	-1.1	76.9	53.7

(*) Missing values: years of schooling = 881; race/color (self-atributed) = 365.

### Characteristics of the Scales

The response rate for the SF-36 was 100%, i.e., all questions were answered by all respondents, despite the fact that 20% of households were replaced due to refusal. However, such units are not sampling losses or selection bias, since the sampling design estimated a surplus of about 25% of cases. The indicator for the quality of understanding of the 15 pairs of questions revealed that only 7.4% showed inconsistency for a single pair of questions, while 7.3% showed inconsistency for 2 to 4 pairs of questions. In the pair of responses that showed the greatest inconsistency (3.7%), respondents claimed both severe limitation of activities such as bathing or dressing and no limitation of vigorous activities. The distribution of items showed that respondents used all categories, with a tendency towards more favourable health status among males aged under 40 and with higher educational level. All scales showed monotonically decreasing gradients with regard to co-morbidities and reported health status (p < 0.05).

The order of the means of item scores within each scale was consistent with the hypothesised expectations (Table 2). In the Physical Functioning scale, the item about vigorous activities (3D) had the lowest mean, and the item about milder activities (3J) had the highest mean. The means decreased over items about functioning ordered in a Guttman scale; for example, a higher frequency of limitations was reported when walking more than 1 km than when walking 100 m. Items in the Physical Functioning scale had the lowest mean scores. The mean scores of items that assessed whether the respondent had accomplished less than he/she would like (physical and emotional aspects) were high, indicating little disability. In the Vitality scale, the mean scores of items that addressed energy (well-being) were higher than the mean scores of items that addressed fatigue. In the Mental Health scale, item 9H (positive aspect of affection) had the highest mean and item 9B (negative aspect of affection) had the lowest mean. The mean score of the item that addressed health transitions was 2.90, which shows that respondents considered that their health was a little better than a year before the interview.

**Table 2:** Mean and confidence intervals (CI 95%) of SF-36 v.2 items. PDSD, 2008

Scale	SF-36 Item	Mean (CI 95%)
*Physical functioning *(PF)	3A. Vigorous activities, such as running, lifting heavy objects, or participating in strenuous sports	2.28 (2.27 - 2.29)
	
	3B. Moderate activities, such as moving a table, pushing a vacuum cleaner, dancing ou swimming	2.48 (2.46 - 2.49)
	
	3C. Lifting or carrying groceries	2.49 (2.47 - 2.50)
	
	3D. Climbing several flights of stairs	2.44 (2.42 - 2.45)
	
	3E. Climbing one flight of stairs	2.54 (2.53 - 2.56)
	
	3F. Bending, kneeling, or stooping	2.50 (2.48 - 2.51)
	
	3G. Walking more than a kilometer	2.49 (2.48 - 2.51)
	
	3H. Walking several hubdreds of meters	2.53 (2.51 - 2.54)
	
	3I. Walking one hundred meters	2.61 (2.60 - 2.62)
	
	3J. Bathing or dressing oneself	2.74 (2.73 - 2.75)

*Role physical *(RF)	4A. Cut down the amount of time one spent on work or other activities	4.11 (4.09 - 4.13)
	
	4B. Accomplished less than you would like	4.06 (4.04 - 4.09)
	
	4C. Limited in kind of work or other activites	4.11 (4.09 - 4.14)
	
	4D. Had difficulty performing work or other activities (i.g., took extra effort)	4.11 (4.09 - 4.13)

*Bodily pain *(BP)	7. Intensity of bodily pain	4.60 (4.58 - 4.63)
	
	8. Extent pain interfered with normal work	5.07 (5.05 - 5.10)

*General health *(GH)	1A. Is your health: excellent, very good, good, fair, poor.	3.05 (3.03 - 3.07)
	
	11A. Seem to get sick a little easier than other people	4.14 (4.12 - 4.16)
	
	11B. As healthy as anybody I know	3.93 (3.91 - 3.96)
	
	11C. Expect my health to get worse	4.05 (4.03 - 4.08)
	
	11D. Health is excellent	3.86 (3.84 - 3.89)

*Vitality *(VT)	9A. Feel full of life	4.10 (4.08 - 4.12)
	
	9E. Have a lot of energy	3.97 (3.95 - 3.99)
	
	9G. Feel worn out	3.93 (3.91 - 3.95)
	
	9I. Feel tired	3.50 (3.48 - 3.52)

*Social functioning *(SF)	6. Extent health problems interfered with normal social activities	4.42 (4.40 - 3.42)
	
	10. Frequency health problems interfered with social activities	4.29 (4.27 - 4.31)

*Role emotional *(RE)	5A. Cut down the amount of time one spent on work or other activities	4.25 (4.23 - 4.27)
	
	5B. Accomplished less than you would like	4.22 (4.20 - 4.24)
	
	5C. Did work or other activities less carefully than usual	4.34 (4.32 - 4.36)

*Mental health *(MH)	9B. Been very nervous	3.53 (3.51 - 3.55)
	
	9C. Felt so down in the dumps that nothing could cheer you up	4.22 (4.20 - 4.24)
	
	9D. Felt calm and peaceful	3.77 (3.75 - 3.79)
	
	9F. Felt downhearted and depressed	4.11 (4.09 - 4.13)
	
	9H. Been happy	4.27 (4.25 - 4.29)

*Health transition*	2.How health is now compared to 1 year ago	2.90 (2.88 - 2.91)

The descriptive and consistency measures for the eight dimensions addressed by the SF-36 are shown in Table 3. All correlations of items with their respective scales exceeded the suggested criterion (r = 0.40) for the internal consistency of items (median = 0.69) and scales, ranging from 0.73 for Social Functioning (SF) and Vitality (VT) to 0.96 for Physical Functioning (PF) and Role Physical (RP). The scales had success rates of 100%, and the smallest difference between the correlations of items with the hypothesised and non-hypothesised scales was 0.10 (9H-MH and 9H-VT), which is more than two standard errors. The General Health, Vitality and Mental Health scales showed the lowest ceiling and floor effects.

**Table 3:** Summary descriptive statistics for the SF-36 v.2 scales. PDSD, 2008 (n = 12.423)

	PF	RP	BP	GH	VT	SF	RE	MH
Reliability*	0.96	0.96	0.84	0.79	0.73	0.73	0.94	0.78
Standard deviation	13.36	11.92	11.68	11.42	11.05	10.48	12.99	12.03
Skewness	-1.10	-1.12	-1.01	-0.87	-0.76	-1.45	-1.42	-0.88
Kurtosis	-0.11	-0.02	-0.09	-0.04	0.10	1.20	0.93	0.30
Ceiling (%)	44.90	53.30	43.30	5.50	13.40	58.50	60.60	14.90
Floor (%)	3.90	3.80	1.40	0.70	0.40	0.70	3.10	0.20
Item internal consistency^#^	0.61-0.87	0.90-0.92	0.72	0.50-0.69	0.48-0.55	0. 58	0.84-0.91	0.47-0.62
Item discriminant validity^&^	0.06-0.50	0.14-0.66	0.18-0.58	0.15-0.46	0.12-0.62	0.23-0.58	0.16-0.66	0.06-0.62

(*) Cronbach alpha coefficient; (#) correlation between items and hypothesized scales corrected for attenuation; (&) correlation between items and other scales; PF - physical functioning, RP - role physical, BP - Bodily pain, GH- general health, VT - vitality, SF- social functioning, RE- role emotional, MH - mental health.

The Physical and Mental Components explained 67.5% of the variance. The correlations between scales in the two dimensions of health showed a pattern that resembles the one described in the literature [7], except for the Role Emotional and Vitality scales, which were strongly correlated with the Physical and Mental Components, respectively, and the Social Functioning scale, which was moderately correlated with both components (Table 4). The Role Physical and Mental Health scales were, respectively, the most valid measures of the Physical and Mental Health Components.

**Table 4:** Hypothesized and observed associations between SF-36 v.2 scales and rotated components. PDSD, 2008 (n = 12.423),

Scale	Hypothesized associations		Correlations with components		Relative validity		Variance explained	
	
	Physical	Mental	Physical	Mental	Physical	Mental	Total	Reliable
*Physical functioning*	0.85	0.17	0.77	0.12	0.83	0.02	0.60	0.63
*Role physical*	0.75	0.44	0.84	0.22	1.00	0.06	0.76	0.79
*Bodily pain*	0.73	0.38	0.62	0.40	0.55	0.21	0.55	0.66
*General health*	0.66	0.48	0.49	0.59	0.34	0.44	0.59	0.74
*Vitality*	0.44	0.73	0.28	0.85	0.11	0.91	0.80	1.00
*Social functioning*	0.52	0.68	0.63	0.49	0.56	0.30	0.64	0.87
*Role emotional*	0.44	0.72	0.75	0.29	0.80	0.11	0.65	0.69
*Mental health*	0.23	0.85	0.16	0.89	0.03	1.00	0.82	1.00

In the comparisons between groups that differed by the presence or absence of depression, subjects who reported having the disease had lower mean scores in all scales (Table 5); the Mental Health scale (MH) discriminated best between the two groups, followed by SF and VT. Among the healthy group and the groups with one, two, or three or more conditions, mean scores decreased as the number of conditions increased. The Bodily Pain, General Health and Vitality scales discriminated best between those groups.

**Table 5:** Mean SF-36 v.2 scale scores (standard error) by mental illness and chronic conditions. PDSD, 2008

		PF	RP	BP	GH	VT	SF	RE	MH
		
	N°	Mean (SE)	Mean (SE)	Mean (SE)	Mean (SE)	Mean (SE)	Mean (SE)	Mean (SE)	Mean (SE)
*Depression*									

Yes	9,975	47.4 (0.1)	48.8 (0.1)	53.4 (0.1)	50.7 (0.1)	58.0 (0.1)	51.0 (0.1)	48.5 (0.1)	51.4 (0.1)
No	1,451	41.9 (0.3)	43.4 (0.3)	45.0 (0.3)	42.5 (0.3)	48.8 (0.3)	42.3 (0.3)	40.1 (0.3)	38.8 (0.3)
F*		251.9	300.5	741.9	779.7	989.0	1005.2	603.9	1603.5
RV		0.16	0.19	0.46	0.49	0.62	0.63	0.38	1.00

*Number of chronic conditions*									

0	4,193	50.4 (0.2)	51.3 (0.2)	57.7 (0.2)	54.2 (0.2)	60.6 (0.2)	53.1 (0.2)	50.6 (0.2)	53.6 (0.2)
1	2,783	47.9 (0.2)	48.8 (0.2)	53.3 (0.2)	50.6 (0.2)	58.1 (0.2)	50.7 (0.2)	48.4 (0.2)	51.0 (0.2)
2	1,842	44.7 (0.3)	47.2 (0.3)	49.8 (0.2)	47.5 (0.2)	55.3 (0.2)	48.8 (0.2)	46.6 (0.3)	48.2 (0.3)
3	2,608	40.8 (0.2)	42.9 (0.2)	44.5 (0.2)	42.8 (0.2)	50.6 (0.2)	44.6 (0.2)	41.9 (0.2)	43.6 (0.2)
F*		324.4	270.0	762.0	590.6	446.0	357.8	233.4	361.7
RV		0.43	0.35	1.00	0.78	0.59	0.47	0.31	0.47

Note: p < 0.0001 for all comparisons; (*) adjusted for age; RV: relative validity; PF - physical functioning, RP - role physical, BP - Bodily pain, GH- general health, VT - vitality, SF- social functioning, RE- role emotional, MH - mental health,

Table 6 summarizes the comparisons between groups according to certain socio-demographic characteristics. The mean scores in all scales were higher in men than in women, and decreased with increasing age. Comparisons according to years of schooling showed that respondents with lower educational level had lower mean scores in all scales. The differences related to age and schooling were statistically significant (p < 0.05). Respondents who self-classified as black reported worse health status in all scales, but these differences were significant only for Role Physical, General Health, Social Functioning, and Role Emotional. The Mental Health, Vitality, and Bodily Pain scales discriminated best between sexes, while the Physical Functioning, Role Physical, and General Health scales discriminated best between groups that differed by age, schooling, and race/colour.

**Table 6:** Mean SF-36 v.2 scale scores (standard error) by age groups, years of schooling and race/color. PDSD, 2008

		PF	RP	BP	GH	VT	SF	RE	MH
		
		Mean (SE)	Mean (SE)	Mean (SE)	Mean (SE)	Mean (SE)	Mean (SE)	Mean (SE)	Mean (SE)
*Sex*									

Male	5,255	48.4 (0.2)	49.3 (0.2)	54.2 (0.2)	50.9 (0.2)	58.8 (0.1)	51.1 (0.1)	48.8 (0.2)	52.0 (0.2)
Female	7,168	45.5 (0.2)	47.1 (0.1)	50.9 (0.1)	48.8 (0.1)	55.3 (0.1)	48.9 (0.1)	46.3 (0.2)	48.1 (0.1)
F adjusted for age		144.5	99.4	260.0	102.9	324.0	146.6	113.3	325.5
p value		<0.01	<0.01	<0.01	<0.01	<0.01	<0.01	<0.01	<0.01
RV		0.44	0.31	0.80	0.32	0.99	0.45	0.35	1.0

*Age groups (years)*									

18-39	3,609	52.4 (0.2)	52.2 (0.2)	56.2 (0.2)	54.0 (0.2)	59.3 (0.2)	52.7 (0.2)	50.9 (0.2)	51.1 (0.2)
40-64	5,647	46.8 (0.2)	48.1 (0.1)	51.7 (0.1)	49.2 (0.1)	56.8 (0.1)	49.8 (0.1)	47.4 (0.2)	49.7 (0.2)
≥ 65	2,170	38.0 (0.3)	42.2 (0.2)	48.6 (0.2)	44.6 (0.2)	54.4 (0.2)	46.1 (0.2)	42.8 (0.3)	49.3 (0.2)
F adjusted for sex		911.0	525.2	344.8	518.8	141.9	296.2	285.1	21.9
p value		<0.01	<0.01	<0.01	<0.01	<0.01	<0.01	<0.01	<0.01
RV		1.00	0.58	0.38	0.57	0.16	0.33	0.31	0.02

*Years of schooling*									

0	1,823	43.9 (0.3)	44.2 (0.3)	49.9 (0.3)	45.7 (0.3)	54.2 (0.3)	47.5 (0.2)	44.0 (0.3)	46.7 (0.3)
1-4	3,363	45.8 (0.2)	47.7 (0.2)	51.6 (0.2)	48.6 (0.2)	56.6 (0.2)	49.5 (0.2)	47.1 (0.2)	49.1 (0.2)
5-8	2,266	47.1 (0.3)	48.5 (0.2)	52.1 (0.2)	49.9 (0.2)	56.9 (0.2)	50.2 (0.2)	47.7 (0.3)	49.9 (0.2)
9-11	2,136	48.7 (0.3)	49.9 (0.2)	54.0 (0.2)	52.0 (0.2)	58.3 (0.2)	51.5 (0.2)	49.2 (0.3)	51.8 (0.3)
≥ 12	1,053	49.6 (0.4)	51.1 (0.3)	54.5 (0.3)	53.7 (0.3)	58.6 (0.3)	51.1 (0.3)	50.0 (0.4)	52.6 (0.4)
F adjusted for age		62.1	77.1	39.5	117.1	39.1	37.2	49.0	56.7
p value		<0.01	<0.01	<0.01	<0.01	<0.01	<0.01	<0.01	<0.01
RV		0.53	0.66	0.34	1.00	0.33	0.32	0.42	0.48

Race/color									

White	5,501	46.9 (0.2)	48.8 (0.1)	52.5 (0.1)	50.5 (0.1)	57.0 (0.1)	50.1 (0.1)	47.9 (0.2)	50.0 (0.2)
Brown	4,360	46.5 (0.2)	47.5 (0.2)	52.1 (0.2)	49.0 (0.2)	57.0 (0.2)	49.8 (0.1)	47.1 (0.2)	49.6 (0.2)
Black	1,248	46.2 (0.3)	47.5 (0.3)	52.3 (0.3)	48.7 (0.3)	56.3 (0.3)	49.4 (0.3)	47.1 (0.3)	49.6 (0.30
F adjusted for age		1.8	19.5	1.5	29.8	1.7	3.6	5.6	1.2
p value		0.16	<0.01	0.21	<0.01	0.17	0.03	<0.01	0.30
RV		0.06	0.65	0.05	1.00	0.06	0.12	0.19	0.04

PF - physical functioning, RP - role physical, BP - Bodily pain, GH- general health, VT - vitality, SF- social functioning, RE- role emotional, MH - mental health.

## Discussion

The findings in this study showed that the psychometric properties of the Brazilian version of the SF-36 (v.2) questionnaire meet the standards established by the IQOLA project [7] Even though the SF-36 had been previously tested in samples of the Brazilian population, this is the first time the Brazilian translation of the questionnaire is used in a nationally representative probability sample.

Data quality was satisfactory, with a high response rate and use of all response categories, suggesting that there were no problems related to the translation of items and categories in the questionnaire. Mean item scores corresponded to the hypothesised scales, except for the Role Physical and Role Emotional scales, due to the change in SF-36 (v.2) questionnaire from binary to ordinal and the consequent increase in the number of response options and categories. The items in the Role Physical scale showed higher mean scores than those found in other studies [19]. These results suggest that the presence of physical and emotional problems in the study population did not lead to significant impairment of daily activities or that, since this is a sensitive question asked by an interviewer, respondents tended not to report that kind of impairment [20].

The reliability estimates exceeded the minimum level (α = 0.70) suggested for comparisons between groups, especially in the case of the Role Physical and Role Emotional scales, which had the highest coefficients and a reduction in ceiling and floor effects. Compared with the estimates in the original version, substantial improvements were noted in item correlations and in the ceiling and floor effects of the Role Physical and Role Emotional scales. All scales exceeded the recommended minimum estimates of internal consistency for group comparisons, but only the Physical Functioning, Role Physical and Role Emotional scales met the criteria for comparisons at the individual level. Even though these effects were still high compared to other scales, their values are similar to those found in studies using the same version of the SF-36 in other countries [6,9]. These improvements, as well the higher sensitivity shown by the Role Physical scale to discriminate between groups that differ by age, schooling, and race/colour, can be attributed to changes in the categorisation of the items that make up these scales.

The correlations between items and their respective scales and the success of scaling were consistent with previous studies [19,21,22]. The correlations between scales and components also showed patterns similar to other studies that used the SF-36, except for the Role Emotional scale, which showed a strong correlation with the Physical Component, in contrast with what was predicted by the model and observed in other studies that used the SF-36 (v.2) [6,9].

In general, construct validity tests showed that PCS scales discriminated better between groups that differed by the presence or absence of chronic diseases, while MCS scales discriminated better between groups that differed by the presence or absence of mental diseases. Men reported better health status than women, age was an important factor related to health, and lower educational levels were associated with poorer health status [23]. Similarly, the percentage of respondents who self-rated their health status as fair or poor was higher among women and increased with age, a pattern also found in the reports of limitation of physical activities and presence of chronic disease. These findings are consistent with the results of previous household surveys of the Brazilian population [14,24,25]. The findings of this study showed that the Brazilian version of the SF-36 (v.2) questionnaire has good discriminatory power between groups of people with or without chronic diseases, suggesting good construct validity. On the other hand, the validity of the Mental Component of the Brazilian version of the SF-36 (v.2) was lower than reported in other studies in view of the lower factor loadings of the Social Functioning and Role Emotional scales used to estimate this component. It has been speculated that cultural and social aspects in developing countries have pivotal role in individual's daily life and may influence the performance of the Social Functioning and Role Emotional scales [26].

## Conclusions

The findings of this study show that the changes made to the SF-36 (v.2) resulted in improved accuracy, reliability, and validity; the study also showed that the Portuguese translation of the questionnaire is adequate, given the completeness of responses and its internal consistency. The results of tests of scaling assumptions support the hypothesised scale structure of the SF-36 questionnaire in Brazil, and the factor loadings obtained can be used to weight the dimensions of the Physical and Mental Components in studies using population samples.

## Competing interests

The authors declare that they have no competing interests.

## Authors' contributions

JL and MRC proposed the article and performed the literature review, data analysis and drafted the first version of the manuscript. CMT, ALN, LAA and MMV drafted the questionnaires and contributed in the analysis and interpretation of the data. All authors read and approved the final manuscript.
